# Effects of Digital-Based Exercise Interventions on Concerns About Falling, Falls Efficacy, and Physical Performance Among Older Adults: Systematic Review and Meta-Analysis

**DOI:** 10.2196/87070

**Published:** 2026-04-30

**Authors:** Zhaojun Wang, Bochi Zhu, Meng Zhou, Xiaojie Xie, Xueyan Zhang

**Affiliations:** 1 School of Nursing, Jilin University, NO. 965 Xinjiang Street, Changchun, Jilin, 130021, China, 86 431 85619596; 2 Department of Neurology, The Second Hospital of Jilin University, Changchun, China

**Keywords:** digital-based, meta-analysis, older adults, concerns about falling, falls efficacy

## Abstract

**Background:**

Falls are prevalent and serious health problems among older adults. Concerns about falling and reduced falls efficacy are common fall-related psychological impairments, representing 2 distinct emotional and cognitive constructs, respectively. The impact of digital-based exercise interventions on these specific constructs remains unclear.

**Objective:**

This systematic review and meta-analysis aimed to synthesize current evidence on digital-based exercise interventions for concerns about falling and falls efficacy among older adults, with a specific focus on determining their differential effects on emotional and cognitive constructs and evaluating their impact on physical performance.

**Methods:**

The PubMed, Web of Science, Cochrane Library, Embase, PsycINFO, CINAHL, CNKI, SinoMed, VIP, and Wanfang databases were systematically searched from their inception dates to May 2025. We searched for published randomized controlled trials on the effects of digital-based interventions on the fear of falling, concerns about falling, and falls efficacy among older adults. The study followed PRISMA (Preferred Reporting Items for Systematic Reviews and Meta-Analyses) guidelines and was performed using Stata 17.0 software (StataCorp LLC).

**Results:**

Eighteen studies involving 2435 participants were included. Meta-analyses revealed significant effects of digital-based exercise interventions on falls efficacy (standardized mean difference 0.70, 95% CI 0.51-0.90; *P*<.001), balance function (mean difference [MD] 4.03, 95% CI 2.57-5.49; *P*<.001), functional mobility (MD −1.65, 95% CI −2.52 to −0.77; *P*<.001), and physical function (MD 0.57, 95% CI 0.12-1.02; *P*=.006) among older adults. However, digital-based exercise interventions had no significant effect on concerns about falling, which is the emotional construct related to falls (standardized MD −0.12, 95% CI −0.28 to 0.05; *P*>.05).

**Conclusions:**

The meta-analysis assessed the efficacy of digital-based exercise interventions on fall-related psychological impairments among older adults and revealed that the effects differed across the constructs. These findings suggest that digital-based exercise interventions have potential benefits for improving falls efficacy and physical performance among older adults compared with controls. However, the effect of digital-based exercise interventions on concerns about falling, which is the emotional construct related to falls, remains uncertain among older adults.

## Introduction

Falling is a prevalent and serious geriatric problem that affects approximately 52% of older adults worldwide [1,2]. The 2022 World Falls Guidelines recommend incorporating the assessment of concerns about falling into multifactorial risk assessments for fall prevention and management [3]. Such fall-related psychological impairments cannot simply be classified as post-fall syndrome, as they can occur in older adults who have never fallen [4]. Instead, they constitute the central mechanism driving the vicious cycle of falls [5,6]. These psychological impairments not only heighten the risk of physical injuries but also impose a significant psychological burden, manifesting as anxiety, depression, and frailty [7-10], and substantially compromise the overall quality of life of older adults. Moreover, the increased risk of falls and related complications can lead to higher mortality rates in older adults [11]. Effective intervention strategies are urgently needed given these profound implications.

To develop effective interventions, a precise understanding of fall-related psychological impairments is crucial. Common fall-related psychological factors include fear of falling, falls efficacy, concerns about falling, and balance confidence, among others [12-14]. Despite the frequent interchangeable use of these terms, they represent different psychological processes with different predictive values for falls [15]. Fear of falling and concerns about falling are both emotional constructs [16]. The former refers to an individual’s emotional response to a real or perceived threat to balance, whereas the latter manifests as a persistent state of worry [17]. On the other hand, falls efficacy and balance confidence are classified as cognitive constructs, primarily referring to an individual’s belief in their own ability or confidence in performing activities without falling. Furthermore, balance confidence is closely related to falls efficacy and is regarded as an important dimension of it [18]. Therefore, we use falls efficacy as an umbrella term to encompass the specific construct of balance confidence. This distinction is methodologically and clinically critical because concerns about falling predict future falls, whereas falls efficacy does not [15]. Combining them in meta-analyses dilutes their unique effects and may be a key source of heterogeneity, potentially obscuring the true efficacy of interventions. Distinguishing between them in research is crucial to avoid this bias and to reveal the specific effects of interventions on different constructs.

Owing to rapid advancements, digital technologies have been increasingly applied to exercise and health management for older individuals. Digital technologies are defined as the use of a series of computing platforms, software, internet-connected devices, and sensors for health care and related purposes, including wearable devices, virtual reality (VR), mobile health apps, and web-based communication platforms [19,20]. Digital-based exercise intervention refers to exercise interventions that use digital technologies to prevent, manage, and treat diseases or promote health. While traditional face-to-face interventions, such as group exercise and physical therapy, have demonstrated efficacy in improving physical function [21-23], they present challenges, including temporal and spatial constraints, health care resource disparities, and high costs [19]. Digital-based exercise interventions may offer novel solutions to these limitations through remote accessibility and automated personalization [24].

While several systematic reviews have investigated interventions for concerns about falling or falls efficacy [12,14], important distinctions set our work apart. First, previous reviews, while covering a broad range of interventions, have not provided a dedicated synthesis of digital-based exercise interventions [12-14]. Our review is distinguished by its exclusive focus on digital interventions and the inclusion of trials up to 2025. This is particularly necessary in the rapidly advancing field of digital interventions, such as those based on VR and smartphone apps, ensuring that our synthesis is current and comprehensive. Second, many syntheses of evidence share a methodological limitation in treating the fear of falling, concerns about falling, or falls efficacy as a single, homogeneous construct [25-27]. This approach contributes to significant heterogeneity and inconsistent findings across systematic reviews, as it fails to capture the differential effects of interventions on cognitive and emotional constructs. To address this, our study explicitly distinguishes cognitive constructs, such as falls efficacy, from emotional constructs, such as concerns about falling. This strategy allows us to reveal the specific intervention effects on each construct, thereby advancing the understanding of their distinct mechanisms in digital-based exercise interventions. Finally, while some reviews have focused on adults of broader ages [28-31], our precise targeting of older adults is designed to yield conclusions with greater specificity and clinical applicability for geriatric care.

This systematic review and meta-analysis aimed to (1) provide an updated synthesis of digital-based exercise interventions for concerns about falling and falls efficacy, (2) determine their specific effects on each distinct construct (cognitive and emotional), and (3) evaluate their impact on physical performance.

## Methods

The study protocol was registered in PROSPERO (CRD42024567108), and the PRISMA (Preferred Reporting Items for Systematic Reviews and Meta-Analyses) guidelines (Checklist 1) were followed [32].

### Search Strategy and Study Selection

The PubMed, Web of Science, Cochrane Library, Embase, PsycINFO, CINAHL, CNKI, SinoMed, VIP, and Wanfang databases were systematically searched for relevant publications. The search terms included Medical Subject Headings (MeSH) and free words related to digital-based interventions, older adults, fear of falling, concerns about falling, falls efficacy, and randomized controlled trials (RCTs). The search terms were combined using Boolean operations. There were no restrictions on the language of the publications. The search period ranged from database inception to May 2025 (the specific search strategies are presented in Multimedia Appendix 1).

Study selection and data extraction were performed independently by 2 researchers. All the retrieved studies were imported into EndNote X20 software (Clarivate) for screening, and duplicate studies were automatically removed by the software. The researchers read the titles, abstracts, and full texts of the studies and subsequently removed studies that did not meet the inclusion criteria. After the study selection was completed, two researchers cross-checked the screening results. Any disagreements were resolved through discussion with a third researcher.

### Eligibility Criteria

#### Inclusion Criteria

The inclusion criteria are summarized in Textbox 1.

Textbox 1. **Inclusion criteria**

- Population: older adults (aged ≥60 y)
- Interventions: participants in the intervention group underwent an exercise intervention that was delivered via digital technologies (wearable devices, exergaming, virtual reality, mobile health apps, and web-based communication platforms).
- Comparison: no digital-based exercise intervention was used in the control group, which included active controls (motor exercise, balance exercise, rehabilitation exercise, and health education) and passive controls (no intervention or maintenance of normal daily living).
- Outcomes**:** studies reporting at least one of the following outcomes.
  - Primary outcome: concerns about falling or falls efficacy included as reported in the original studies, whether as a primary or secondary outcome. Concerns about falling was measured by validated scales (Falls Efficacy Scale–International, Icon-Falls Efficacy Scale, and Short Falls Efficacy Scale). Falls efficacy was measured by validated scales (Falls Efficacy Scale, Activities-Specific Balance Confidence Scale, and Modified Falls Efficacy Scale).
  - Secondary outcome: physical performance included measures of balance (Berg Balance Scale), functional mobility (Timed Up and Go Test), and physical function (Short Physical Performance Battery).
- Study design: randomized controlled trial

#### Exclusion Criteria

We excluded studies that focused primarily on individuals diagnosed with dementia, duplicate studies, studies with unavailable full texts or important data, meeting abstracts, study protocols, studies without treatment details or primary outcomes, pilot studies, and feasibility trials.

### Data Extraction

Two researchers independently extracted data from the complete original studies using a uniform data extraction template. The data extracted included the author’s name, country, year of publication, study population, age, sex, sample size, intervention, control, intervention period, duration of intervention, frequency of intervention, outcome indicators, end values in the control and intervention groups, and evaluation tools for the outcome indicators. Any discrepancies between the initial disagreements were first discussed between the 2 researchers until a consensus was reached. If an agreement could not be reached, the disagreement was referred to a third, senior researcher for adjudication and a final decision.

### Assessment of the Risk of Bias and Certainty of the Evidence

An independent appraisal of the risk of bias for each included study was performed by 2 investigators who used the Cochrane Risk of Bias 2 (RoB2) instrument. The appraisal covered 5 predefined domains: randomization procedure, deviations from intended interventions, missing outcome data, measurement of the outcome, and selection of the reported result. On the basis of the assessments across these domains, the researchers subsequently assigned an overall risk judgment of “low risk,” “some concerns,” or “high risk” to each included study.

We evaluated the certainty of the evidence using the Grading of Recommendations Assessment, Development, and Evaluation (GRADE) Profiler Guideline Development Tool. In accordance with the GRADE system, the certainty of evidence for each outcome was classified as high, moderate, low, or very low. Any disagreements were referred to a third researcher for adjudication.

### Data Analysis

Statistical analysis was conducted using Stata 17.0 (StataCorp LLC). The outcome indicators were continuous variables, and effect sizes were expressed as the means and SDs combined with 95% CIs to report the results of the continuous variable data analysis. For continuous variables, the mean difference (MD) was selected when the same outcome indicator was measured on the same scale; otherwise, the standardized mean difference (SMD) was adopted. Between-group heterogeneity of synthetic effect sizes was tested using the *I*^2^ statistic and *P* value. Random-effects models were used for the meta-analysis because of anticipated heterogeneity. To further explore the factors influencing the intervention, additional subgroup analyses were performed on the basis of the setting, intervention qualifications, intervention duration, exercise mode, digital platform, duration of the intervention, exposure dosage, and scale-scoring method. Reverse scoring scale–converted data (mean x−1, with the SD unchanged) were included in the analysis alongside positive scoring scale data to improve the accuracy and reliability of the results. For multiarm RCTs, this review included only comparisons that involved an eligible digital-based exercise intervention group and a control group. In the meta-analysis, each trial contributed only one comparison pair to prevent double-counting of control group data. To assess the robustness of the combined effect sizes, sensitivity analyses were performed using a remove-one-article method. For outcomes with data from more than 10 studies, publication bias was assessed via funnel plots [33]. The Egger test [34] and the Begg test [35] were used to further assess publication bias.

## Results

### Search Results

**Figure 1.**
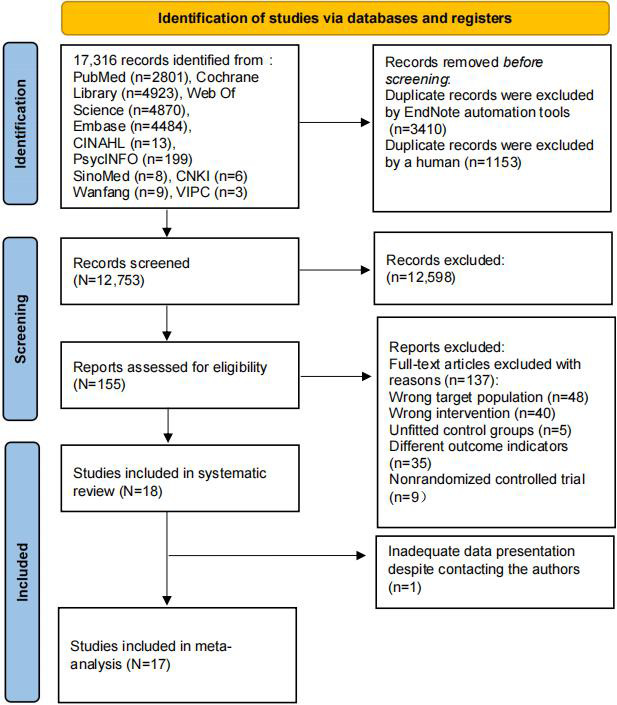
PRISMA (Preferred Reporting Items for Systematic Reviews and Meta-Analyses) flow diagram of the search process.

In total, 17,316 articles were retrieved from 10 databases; 3410 duplicates were removed by EndNote X20 software, and 1153 duplicates were removed manually. A total of 12,598 articles were excluded because their titles and abstracts were not relevant to the aim of the study. After the full texts were reviewed, 137 studies were excluded. Ultimately, 18 studies [36-53] were included in this study. One study [44] was excluded from the quantitative synthesis because of inadequate data presentation despite contacting the authors. Therefore, 17 studies were included in the meta-analysis (Figure 1).

### Characteristics of the Studies

The characteristics of the included studies are presented in Table 1. A total of 2435 older adults were included, with average ages ranging from 67.8 (SD 2.98) to 84.1 (SD 5.5) years. The proportion of women varied between 26.7% and 100%. The publication years ranged from 2013 to 2025. These studies were conducted in the following countries: Australia (n=2) [39,51], China (n=3) [40,42,47], Korea (n=2) [36,53], Switzerland (n=1) [49], Spain (n=2) [41,50], Iran (n=1) [45], Singapore (n=1) [37], Italy (n=1) [38], Türkiye (n=1) [46], the Netherlands (n=1) [48], Pakistan (n=1) [43], Chile (n=1) [52], and Brazil (n=1) [44]. The study settings included a range of environments, such as communities [37,41,46,48,51-53], hospitals [38,39,43,44,47,49,50], and nursing homes or welfare centers [36,40,42,45].

The intervention details of the included studies are presented in Table 2. The interventions were primarily delivered by qualified health care professionals [37-40,42-44,48-50], with physiotherapists being the most common. In other cases, the delivery was managed by trained nonspecialists [36,41,51,52], including research assistants or physical education instructors. However, the specific qualifications of the interveners were not reported in several trials [45-47,53]. The digital-based exercise modes included balance-dominant balance training [38,41,44,45,48,51]; balance-inclusive multimodal training, which combines balance with one or more forms of resistance or aerobic exercise; and nonbalance training dominated by aerobic exercise [52]. Among these, balance-inclusive multimodal training was the most common. Furthermore, predesigned programs such as Baduanjin [42] and the Otago Exercise Program [47] incorporate various elements, including balance, strength, and flexibility, and are classified as balance-inclusive multimodal training. Exergaming [36-41,43-45,51-53] was the most frequently used digital delivery platform for exercise interventions, followed by computer-based applications [46,47,49,50], while only 2 studies [42,48] used VR to provide a virtual environment for exercise. The duration of individual interventions ranged from 30 to 120 minutes per session. The most frequently implemented duration for a single session was 60 minutes. The frequency of interventions ranged from 1 to 5 times per week, and the most commonly prescribed intervention frequency ranged from 2 to 3 times per week. The duration ranged from 2 weeks to 12 months, and the most common duration was 8 weeks.

The outcomes included concerns about falling, falls efficacy, balance, functional mobility, and physical function. A wide variety of measurement scales have been applied among studies. Nine studies [38,40,41,43,48-52] used the Falls Efficacy Scale–International (FES-I) scale, Short Falls Efficacy Scale, or Iconographical Falls Efficacy Scale, which reflect concerns about falling, and 9 studies [36,37,39,42,44-47,53] used the Activities-Specific Balance Confidence Scale, Falls Efficacy Scale, or Modified Falls Efficacy Scale (MFES), which reflect balance confidence or falls efficacy. Among the included studies, the MFES and Activities-Specific Balance Confidence Scale are positive scoring scales, where higher scores indicate greater falls efficacy. In contrast, the Falls Efficacy Scale is a reverse scoring scale, meaning that higher scores reflect lower falls efficacy. The FES-I, Short Falls Efficacy Scale, and Iconographical Falls Efficacy Scale also follow a positive scoring direction, but in their case, higher scores indicate a greater level of concerns about falling. The Berg Balance Scale [36,38,45,47,52,53], Timed Up and Go Test [36,37,40,43,45,47,49,51-53], and Short Physical Performance Battery [39,46,49-51] were used to evaluate balance, functional mobility, and physical function, respectively. Only 3 out of the 18 (16.7%) included studies [37,43,47] reported concerns about falling or falls efficacy as their primary outcome. Seven (38.9%) studies [39,40,44,48-51] explicitly defined it as a secondary outcome, whereas the remaining studies (44.4%) [36,38,41,42,45,46,52,53] did not clearly distinguish between primary and secondary outcomes in their original texts.

**Table 1.** Characteristics of the included studies (N=18).

Study (author, year)	Participant characteristics						Primary outcome (falls efficacy)
	Country	Participants	Setting	Age (y)	Total sample size	Female (%)	
Lee and Shin [36] (2013)	Korea	Older adults with diabetes mellitus	Welfare center	CG^a^: 74.29 (5.20)^b^ IG^c^: 73.78 (4.77)^b^	55	70.9	MFES^d^
Kwok and Pua [37] (2016)	Singapore	Older adults with mild-to-moderate physical frailty	Community	CG: 69.8 (7.5)^b^ IG: 70.5 (6.7)^b^	80	85	MFES
Morone et al [38] (2016)	Italy	Older women with bone loss	Hospital	CG: 70.05 (4.93)^b^ IG: 67.8 (2.98)^b^	36	100	Short-FES-I^e^
van den Berg et al [39] (2016)	Australia	Older adults	Hospital	CG: 82 (13)^b^ IG: 78 (10)^b^	56	62.1	FES^f^
Liao et al [40] (2019)	China	Frail older adults	Daycare centers	CG: 84.1 (5.5)^b^ IG: 79.6 (8.5)^b^	52	69.2	FES-I^g^
Montero-Alía et al [41] (2019)	Spain	Older adults	Community	CG: 75.4 (72.7‐78.6)^h^ IG: 75.1 (72.6‐78.7)^h^	608	59.1	Short-FES-I
Sun et al [42] (2020)	China	Older adults with osteoporosis	Nursing institution	CG: 72.53 (7.04)^b^ IG: 73.96 (6.32)^b^	60	75.1	MFES
Zahedian-Nasab et al [45] (2021)	Iran	Older adults with fall risk	Nursing homes	CG: 72 (7.81)^b^ IG: 69.67 (7.73)^b^	60	26.7	FES
Khushnood et al [43] (2021)	Pakistan	Older adults	Hospital	N/A^i^	83	38.5	ABC^j^
Rebêlo et al [44] (2021)^k^	Brazil	Older adults with balance disorders	Hospital	CG: 71.41 (5.94)^b^ IG: 69.25 (5.67)^b^	37	83.8	FES-I
Yang et al [47] (2022)	China	Older people with fear of falling	Hospital	CG: 69.60 (3.21)^b^ IG: 69.92 (2.15)^b^	100	55	MFES
Tekin and Cetisli-Korkmaz [46] (2022)	Türkiye	Older adults	Community	C: 70.34 (5.37)^b^ I: 68.34 (4.33)^b^	255	69.4	MFES
Gerards et al [48] (2023)	Netherlands	Older adults	Community	CG: 73 (8)^b^ IG: 73 (10)^b^	82	79.3	FES-I
Lee [53] (2023)	Korea	Older adults	Community	CG: 79.10 (3.90)^b^ IG: 80.39 (2.57)^b^	57	45.6	MFES
Sturnieks et al [51] (2024)	Australia	Older adults	Community	CG: 72.5 (5.5)^b^ IG: 72.6 (5.7)^b^	507	71	Icon-FES^l^
Hager et al [49] (2024)	Switzerland	Older people at risk of falling	Community	CG: 79 (6.6)^b^ IG: 79 (7.0)^b^	172	74	FES-I
Prieto-Moreno et al [50] (2024)	Spain	Older adults with hip fracture	Hospital	CG: 80.07 (7.74)^b^ IG: 79.55 (7.11)^b^	105	71.4	Short-FES-I
Vásquez-Carrasco [52] (2025)	Chile	Older females	Community	CG: 76.2 (2.03)^b^ IG: 72.2 (6.76)^b^	30	100	FES-I

a CG: control group.

b Mean (SD).

c IG: intervention group.

d MFES: Modified Fall Efficacy Scale.

e Short-FES-I: Short Falls Efficacy Scale International.

f FES: Falls Efficacy Scale.

g FES-I: Falls Efficacy Scale International.

h Medium (IQR).

i Not applicable.

j ABC: Activities-Specific Balance Confidence scale.

k Included in the qualitative synthesis but not in the quantitative synthesis.

l Icon-FES: Iconographical Falls Efficacy Scale.

**Table 2.** Intervention details of the included studies (N=18).

Study	Qualifications of interveners	Intervention	Control	Exercise mode	Digital platform	Duration for a single session	Exposure
Lee and Shin [36] (2013)	Research assistant	VR^a^ exercise + health education on diabetes management	Health education on diabetes management	Balance + resistance + aerobic training	Exergaming	50 minutes	Twice a week, for 10 weeks
Kwok and Pua [37] (2016)	Physiotherapist and a therapist assistant	Nintendo Wii exercise program	Gym-based exercise intervention	Balance + resistance training	Exergaming	60 minutes	Once a week, for 12 weeks
Morone et al [38] (2016)	Physiotherapist	Balance training based on Nintendo WiiFit	Routine balance training	Balance training	Exergaming	60 minutes	Twice a week, for 8 weeks
van den Berg et al [39] (2016)	A physiotherapist and a therapist assistant	Computer-based interactive exercises + routine rehabilitation nursing	Routine rehabilitation nursing	Balance + resistance training	Exergaming	60 minutes	Five times a week, for 2 weeks
Liao et al [40] (2019)	Experienced physical therapist	Kinect-based exergaming exercise	Combined exercise training	Tai chi + balance + resistance + aerobic training	Exergaming	60 minutes	Three times a week, for 12 weeks
Montero-Alía et al [41] (2019)	Monitors who had received standardized training	Balance training based on Nintendo Wii game	Routine care	Balance training	Exergaming	30 minutes	Twice a week, for 3 months
Sun et al [42] (2020)	Therapist	VR-based Baduanjin training + conventional osteoporosis treatment	Conventional osteoporosis treatment	Baduanjin training	VR	50 minutes	Three times a week, for 12 months
Zahedian-Nasab et al [45] (2021)	N/A^b^	VR balancing exercise based on Xbox Kinect	Routine activities	Balance training	Exergaming	30‐45 minutes	Twice a week, for 6 weeks
Khushnood et al [43] (2021)	Therapist	VR-based WiiFit exercise	Conventional balance training exercises	Balance + aerobic training	Exergaming	30 minutes	Twice a week, for 8 weeks
Rebêlo et al [44] (2021)^c^	Therapist	Balance training based on immersive VR	Conventional body balance training	Balance training	Exergaming	50 minutes	Twice a week, for 8 weeks
Yang et al [47] (2022)	N/A	Internet-based Otago Exercise Program workouts	Health education	Balance + resistance training	Application	30 minutes	Three times a week, for 6 months
Tekin and Cetisli-Korkmaz [46] (2022)	N/A	Internet-based calisthenic exercise	No intervention	Resistance + aerobic training	Application	Six different types of calisthenic exercises (repeated 10 times)	Five times a week, for 4 weeks
Gerards et al [48] (2023)	Physiotherapist	Perturbation-based balance training + usual care	Usual care	Balance training	VR	30 minutes	Once a week, for 3 weeks
Lee [53] (2023)	N/A	The home-based exergame program + health education	Health education	Balance + resistance training	Exergaming	50 minutes	Three times a week, for 8 weeks
Sturnieks et al [51] (2024)	Exercise science graduates	Computerized game-based sports training	Health education	Balance training	Exergaming	120 minutes	Once a week, for 12 months
Hager et al [49] (2024)	Physiotherapist	Partially supervised exercise	Otago exercise program	Balance + resistance training	Application	30‐45 minutes	Three times a week, for 12 months
Prieto-Moreno et al [50] (2024)	Physiotherapist or occupational therapist	ActiveHip intervention based on mHealth^d^ + usual rehabilitation	Usual rehabilitation	Balance + resistance training	Application	One video-recorded session	Three times a week, for 3 months
Vásquez-Carrasco et al [52] (2025)	Trained physical education instructor	Xbox Kinect Sports	Usual daily routines	Aerobic training	Exergaming	60 minutes	Three times a week, for 24 weeks

a VR: virtual reality

b Not applicable.

c Included in the qualitative synthesis but not in the quantitative synthesis.

d mHealth: mobile health.

### Risk of Bias in RCTs

**Figure 2.**
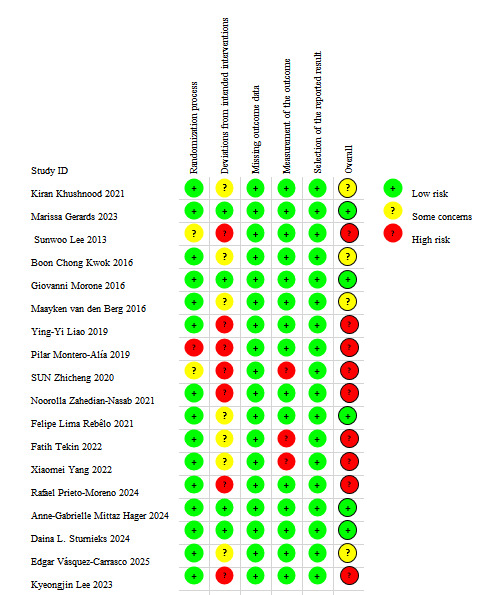
Risk of bias summary [36-53].

The risk of bias was assessed for the 18 RCTs included. The majority of studies were judged to have a high risk of bias or to raise some concerns overall. For the domain of the randomization process, the majority of studies were judged to be at low risk of bias. Two studies were rated as having some concerns due to a lack of information on allocation concealment. One study was assessed as being at high risk because significant baseline differences suggested potential problems with the randomization procedure. For deviations from intended interventions, most studies were assessed as high risk or with some concerns, primarily attributed to a lack of blinding for participants and intervention providers. For the domain of outcome measurement, 3 studies were assessed as high risk because blinding of the outcome assessors was not implemented. The risk of bias was assessed as low across all remaining domains (Figure 2).

### Effectiveness of Interventions

#### Concerns About Falling

**Figure 3.**
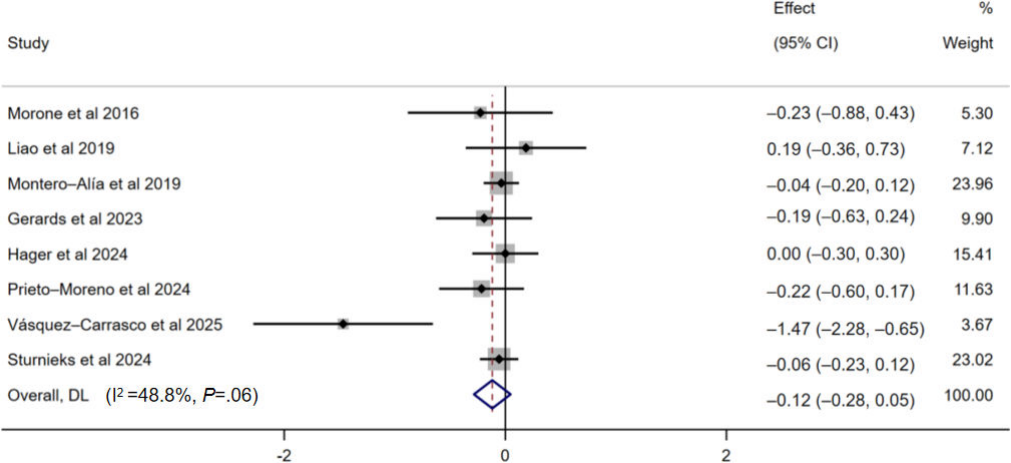
Forest plot showing the effects of digital-based exercise interventions on concerns about falling (n=8; lower scores indicate greater improvement) [38,40,41,48-52]. Weights are from a random-effects model.

A total of 8 studies evaluated the impact of digital-based exercise interventions on concerns about falling in older adults, involving 758 participants in the intervention groups and 834 in the control groups. The pooled results from 8 studies revealed that digital-based exercise interventions had a significant effect on concerns about falling (SMD −0.12, 95% CI −0.28 to 0.05; *P*>.05; *I*²=48.8%; low certainty of the evidence; Figure 3). None of the subgroup analyses yielded statistically significant results. Although the point estimates varied, none of the tests for subgroup differences were significant (*P*>.05), with 1 exception: the exercise mode subgroup showed a nominally significant difference (*P*=.003). However, this finding was driven entirely by a single study in the nonbalance training subgroup, which reported a statistically significant reduction in concerns about falling (SMD −1.47, 95% CI −2.28 to −0.65). When this single study was excluded, the difference between the remaining exercise mode subgroups was no longer significant (*P*=.86), indicating that the initial subgroup difference was not robust. Therefore, there is no clear evidence to identify which intervention characteristics are most effective for reducing concerns about falling (Table 3).

**Table 3.** Subgroup analysis of digital-based exercise interventions on concerns about falling and falls efficacy.

Outcome and variable	Numbers of comparisons	Meta-analysis results, SMD^a^ (95% CI)	Heterogeneity	Heterogeneity between groups (*P* value)
Concerns about falling				
Setting				.45
Community	5	−0.14 (−0.36 to 0.08)	*I*²=66.9%, *P*=.02	
Senior care facilities	1	0.19 (−0.36 to 0.73)	—^b^	
Hospital	2	−0.22 (−0.55 to 0.11)	*I*²=0%, *P*=.98	
Qualifications of interveners				.29
Nonprofessional physiotherapist	2	−0.05 (−0.16 to 0.07)	*I*²=0%, *P*=.87	
Physiotherapist	6	−0.22 (−0.53 to 0.09)	*I*²=60.7%, *P*=.03	
Exercise mode				*.003*
Balance-inclusive multimodal training	3	−0.04 (−0.26 to 0.18)	*I*²=0%, *P*=.46	
Balance training	4	−0.06 (−0.17 to 0.05)	*I*²=0%, *P*=.88	
Nonbalance training	1	−1.47 (−2.28 to −0.65)	—	
Digital platform				.88
Exergaming	5	−0.15 (−0.42 to 0.11)	*I*²=68.3%, *P*=.01	
VR^c^	1	−0.19 (−0.63 to 0.24)	—	
Application	2	−0.08 (−0.32 to 0.15)	*I*²=0%, *P*=.39	
Duration of the intervention (wk)				.67
<12	2	−0.20 (−0.57 to 0.16)	*I*²=0%, *P*=.93	
≥12	6	−0.11 (−0.31 to 0.09)	*I*²=62%, *P*=.02	
Exposure dosage				.46
<20	2	−0.20 (−0.57 to 0.16)	*I*²=0%, *P*=.93	
20‐50	3	−0.05 (−0.19 to 0.10)	*I*²=0%, *P*=.48	
>50	3	−0.31 (−0.78 to 0.17)	*I*²=82.7%, *P*=.003	
Falls efficacy				
Setting				.34
Community	3	0.55 (0.30 to 0.80)	*I*²=24.1%, *P*=.27	
Senior care facilities	3	0.84 (0.53 to 1.15)	*I*²=0%, *P*=.87	
Hospital	3	0.77 (0.27 to 1.27)	*I*²=71.1%, *P*=.03	
Qualifications of interveners				.51
Nonprofessional physiotherapist	5	0.75 (0.57 to 0.93)	*I*²=0%, *P*=.76	
Physiotherapist	4	0.59 (0.15 to 1.03)	*I*²=69.8%, *P*=.02	
Exercise mode				.46
Balance-inclusive multimodal training	8	0.69 (0.48 to 0.89)	*I*²=42.8%, *P*=.09	
Balance training	1	0.90 (0.37 to 1.43)	—	
Digital platform				.99
Exergaming	6	0.69 (0.37 to 1.00)	*I*²=57.5%, *P*=.04	
VR	1	0.73 (0.21 to 1.25)	—	
Application	2	0.71 (0.48 to 0.93)	*I*²=26.5%, *P*=.24	
Duration of the intervention (wk)				.60
<12	6	0.75 (0.51 to 0.98)	*I*²=37.2%, *P*=.16	
≥12	3	0.62 (0.23 to1.02)	*I*²=55.9%, *P*=.10	
Exposure dosage				.72
<20	4	0.63 (0.16 to 1.10)	*I*²=72.5%, *P*=.01	
20‐50	3	0.69 (0.48 to 0.90)	*I*²=0%, *P*=.68	
>50	2	0.83 (0.50 to 1.15)	*I*²=0%, *P*=.64	
Scale-scoring method				.62
Positive scoring	7	0.73 (0.53 to 0.94)	*I*²=34.3%, *P*=.17	
Reverse scoring	2	0.56 (−0.10 to 1.23)	*I*²=68.3%, *P*=.08	

a SMD: standardized mean difference.

b Not applicable.

c VR: virtual reality.

The sensitivity analysis for the cognitive constructs demonstrated that the overall results were robust, as no single study significantly altered the pooled effect estimate. The sensitivity analysis of affect-based constructs revealed that a study by Vásquez-Carrasco et al [52] (SMD −1.47, 95% CI −2.28 to −0.65) was an outlier. After this study was excluded, the intervention effects remained nonsignificant (SMD −0.06, 95% CI −0.16 to 0.04; *P*>.05; *I*²=0.0%).

#### Falls Efficacy

**Figure 4.**
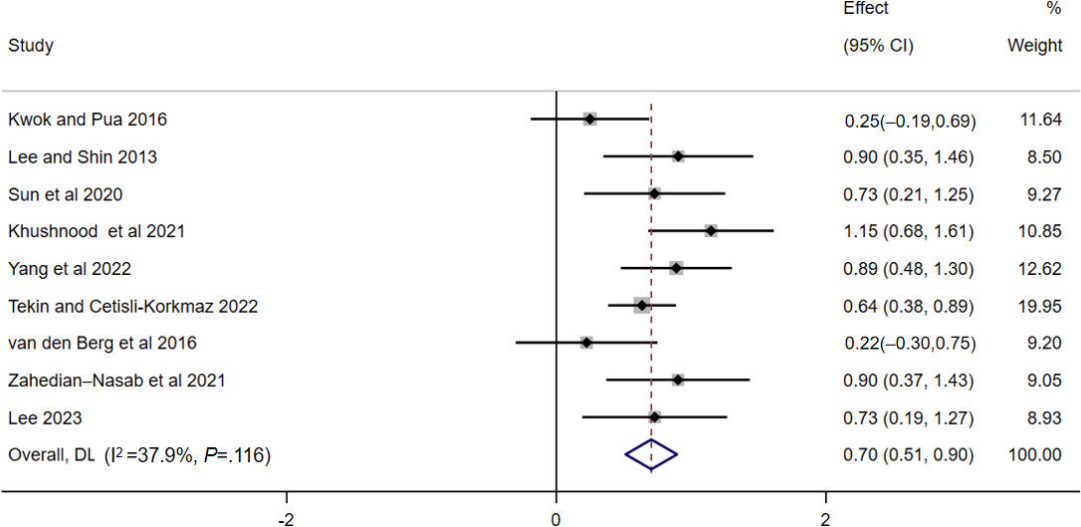
Forest plot showing the effects of digital-based exercise interventions on falls efficacy (n=9; higher scores indicate greater improvement) [36,37,39,42,43,45-47,53].

The meta-analysis of 9 studies revealed that digital-based exercise interventions significantly improved falls efficacy among older adults (SMD 0.70, 95% CI 0.51-0.90; *P*<.001; *I*²=37.9%; moderate certainty of the evidence; Figure 4). With respect to intervention setting, the effect size was greatest for senior care facilities (SMD 0.84, 95% CI 0.53-1.15), followed by hospitals (SMD 0.77, 95% CI 0.27-1.27), and then community settings (SMD 0.55, 95% CI 0.30-0.80), although the difference between groups was not significant (*P*=.34). With respect to exercise mode, balance training had a greater effect (SMD 0.90, 95% CI 0.37-1.43) than multimodal training did (SMD 0.69, 95% CI 0.48-0.89), but the difference was not statistically significant (*P*=.46). Similarly, a higher exposure dosage was associated with a greater effect, with the >50 times subgroup (SMD 0.83, 95% CI 0.50-1.15) having a greater effect than the <20 times (SMD 0.63, 95% CI 0.16-1.10) and 20 to 50 times (SMD 0.69, 95% CI 0.48-0.90) subgroups did, and there was no significant between-group difference (*P*=.72). With respect to other subgroup variables, including the intervention qualifications, digital platform type, intervention duration, and scale-scoring method, the effect sizes were similar across categories (SMD range 0.56‐0.90), with no significant differences between the subgroups (all *P*>.05; Table 3).

The sensitivity analysis for the falls efficacy demonstrated that the overall results were robust, as no single study significantly altered the pooled effect estimate.

#### Balance (Berg Balance Scale)

**Figure 5.**
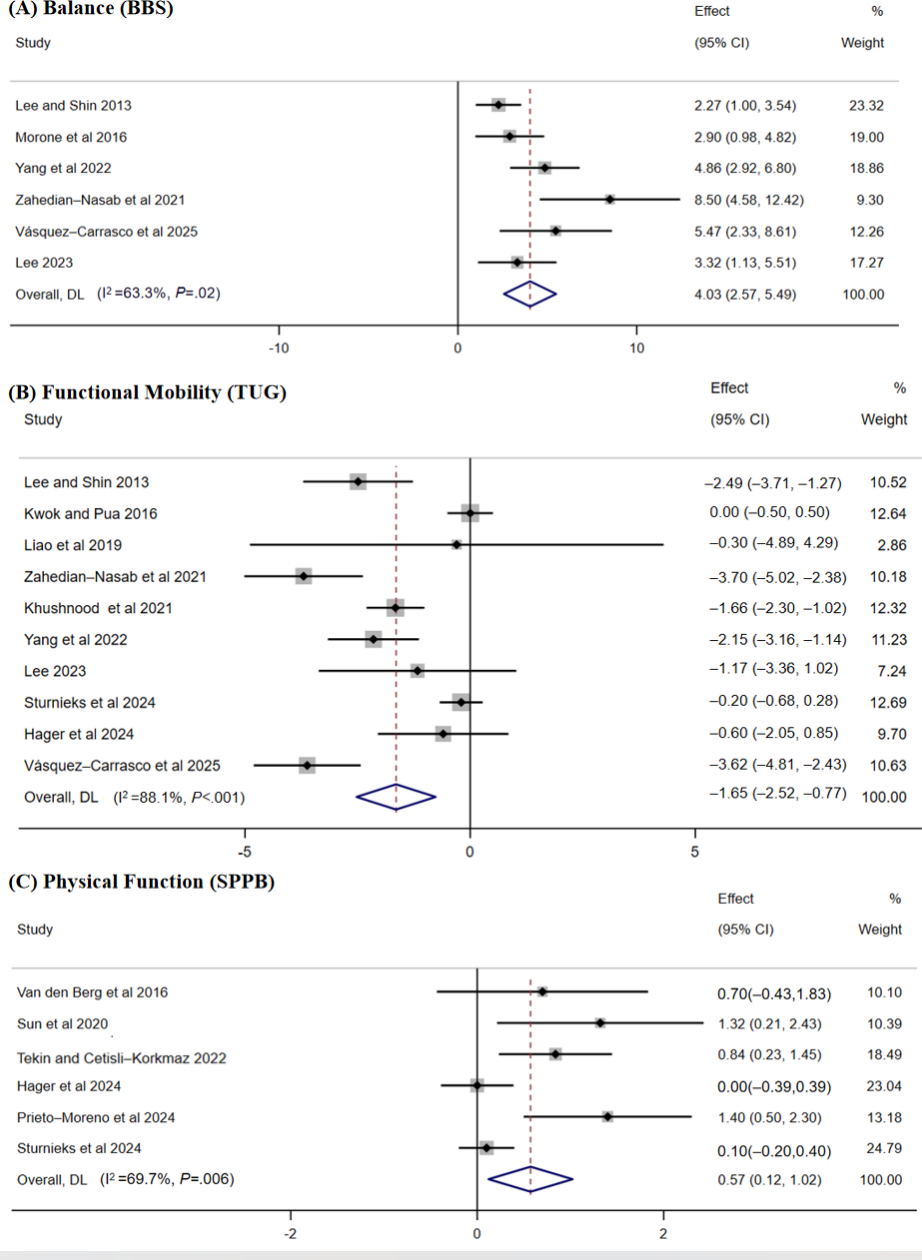
Forest plot showing the effects of digital-based exercise interventions on (A) balance (Berg Balance Scale [BBS]), (B) functional mobility (Timed Up and Go Test [TUG]), and (C) physical function (Short Physical Performance Battery [SPPB]). A lower TUG score indicates a more significant improvement in functional mobility, whereas higher BBS and SPPB scores indicate more significant improvements in balance and physical function, respectively [36-40,42,43,45-47,49-53].

The pooled results from 6 studies [36,38,45,47,52,53] revealed a significant effect of digital-based exercise interventions on balance (MD 4.03, 95% CI 2.57-5.49; *P*<.001; *I*²=63.3%; moderate certainty of the evidence; Figure 5A). With respect to balance, most subgroups, including the intervention setting, intervention qualifications, exercise mode, and intervention duration showed no significant differences between subgroups (*P*>.05). The only statistically significant subgroup differences were found between the exposure dosage subgroups (*P*=.04). An exposure dosage of less than 20 times (MD 5.43, 95% CI −0.03 to 10.89) had a greater effect than an exposure dosage of more than 50 times (MD 5.03, 95% CI 3.38-6.68) and 20 to 50 times (MD 2.53, 95% CI 1.43-3.63; Table S1 in Multimedia Appendix 1).

Sensitivity analysis revealed that no individual study was able to change the results, but the heterogeneity decreased from 63.3% to 42.6% when Zahedian-Nasab’s [45] study was excluded (MD 3.46, 95% CI 2.30-4.61; *P*<.001; *I*²=42.6%).

#### Functional Mobility (Timed Up and Go Test)

In ten studies [36,37,40,43,45,47,49,51-53], researchers reported functional mobility. The meta-analysis indicated a significant effect of digital-based exercise interventions on functional mobility compared with the control group; however, considerable heterogeneity was noted among the included studies (MD −1.65, 95% CI −2.52 to −0.77; *P*<.001; *I*²=88.1%; moderate certainty of the evidence; Figure 5B). Subgroup analyses for functional mobility were performed across the intervention setting, exercise mode, the duration of intervention, digital platform, the qualifications of interveners, and exposure dosage. With respect to exercise mode, subgroup differences were initially observed (*P*=.01), with nonbalance training yielding the greatest effect size (MD −3.62, 95% CI −4.81 to −2.43) compared with balance-inclusive multimodal training (MD −1.29, 95% CI −2.22 to −0.37) and balance training (MD −1.89, 95% CI −5.32 to 1.53). However, the significant subgroup difference disappeared (*P*=.74) after the single study of nonbalance training was excluded. With respect to intervention setting, the digital-based exercise intervention delivered in senior care facilities (MD −2.89, 95% CI −4.11 to −1.68) showed a greater improvement effect than that in community settings (MD −1.03, 95% CI −1.20 to 0.07) and hospitals (MD −1.80, 95% CI −2.34 to −1.26), although the difference between groups was not statistically significant (*P*=.08). Interventions lasting less than 12 weeks (MD −2.30, 95% CI −3.33 to −1.28) improved functional mobility more strongly than interventions lasting 12 weeks or more did (MD −1.20, 95% CI −2.28 to −0.11; *P*=.15). For other subgroup variables, including the qualifications of the intervener, digital platform type, and exposure dosage, the effect sizes were similar across categories (MD range: −2.08 to −1.29), with no significant differences between the subgroups (all *P*>.05; Table S1 in Multimedia Appendix 1). Sensitivity analysis revealed that no individual study was able to change the results.

#### Physical Function (Short Physical Performance Battery)

In 6 studies [39,42,46,49-51], researchers reported the effects of digital-based exercise interventions on physical function. The meta-analysis revealed that digital-based exercise interventions significantly improved physical function among older adults (MD 0.57, 95% CI 0.12-1.02; *P*=.006; *I*²=69.7%; moderate certainty of the evidence; Figure 5C). The hospital subgroup had the greatest effect size (MD 1.13, 95% CI 0.42-1.83), which was greater than that of the community subgroup (MD 0.24, 95% CI −0.16 to 0.64; *I*²=64.5%; *P*=.060). After the senior care facility subgroup (only 1 study) was excluded, significant between-group differences were observed (*P*=.03). The effect size was greater for the application subgroup (MD 0.67, 95% CI −0.14 to 1.49) than for the exergaming subgroup (MD 0.14, 95% CI −0.16 to 0.44; *I*²=81.5%; *P*=.005). However, after excluding the VR subgroup (only 1 study), the difference between groups was no longer statistically significant (*P*=.23). The 20-50 times subgroup yielded the strongest effect (MD 1.02, 95% CI 0.51-1.53), which was superior to that of the >50 times subgroup (MD 0.21, 95% CI −0.22 to 0.64; *I*²=59.1%; *P*=.09). After the <20 times subgroup (only 1 study) was excluded, significant between-group differences were observed (*P*=.02). The intervention duration and qualifications of the interveners showed no significant between-group differences (*P*=.43 and *P*=.52, respectively; Table S1 in Multimedia Appendix 1). Sensitivity analysis revealed that no individual study was able to change the results.

#### Publication Bias

For the outcome of functional mobility, the funnel plot appeared asymmetrical; however, both the Egger test (*P*=.09) and the Begg test (*P*=.53) indicated no statistically significant publication bias (Figure S1 in Multimedia Appendix 1).

#### Narrative Synthesis

One study [44] was excluded from the quantitative synthesis because of inadequate data presentation, despite attempts to contact the authors. This study revealed that immersive VR-based balance training improved older adults’ mobility (Timed Up and Go Test: MD −1.71, 95% CI −2.73 to −0.69) but did not alleviate concerns about falling (FES-I: MD 1.80, 95% CI −2.02 to 5.62), with no significant differences compared with the control group.

## Discussion

### Effect of Digital-Based Exercise Interventions on Falls Efficacy and Concerns About Falling

The meta-analysis assessed the efficacy of digital-based exercise interventions on concerns about falling and falls efficacy among older adults. Overall, the interventions were effective at improving falls efficacy but not concerns about falling, the emotional construct related to falls. This key distinction suggests that the cognitive and emotional constituents may represent 2 relatively independent intervention targets, requiring distinct strategies to address each effectively.

For falls efficacy, Gao et al [54] reported nonsignificant effects of VR interventions on falls efficacy measured by the MFES in nondisabled older adults. This difference may be because, compared with previous studies, this analysis pooled data from more studies, thus improving the statistical power and stability. The improvement in cognitive constructs through digital-based exercise interventions may be attributed to their ability to provide structured, repetitive exercises and feedback. These findings align with substantial evidence indicating that physical exercise directly enhances physical function and balance [55,56], thereby increasing self-efficacy. This process can be attributed to the underlying neurobiological mechanism: the multisensory stimulation (eg, visual, auditory, and vestibular) provided by digital exercise interventions promotes plasticity in the central nervous system, thereby enhancing the ability of the brain to process sensory information and maintain postural control [57-59]. Consequently, the experience of successfully completing motor challenges, reinforced directly through this neural reshaping, strengthens older adults’ balance confidence or falls efficacy. Subgroup analyses explored the potential moderating effects of various intervention characteristics on falls efficacy, and no statistically significant between-group differences were found across all the subgroups, indicating that the improvement effect of digital-based exercise interventions on falls efficacy was consistent across different characteristics. For intervention setting, the effect size was the greatest in senior care facilities, followed by hospitals and then community settings, which may be attributed to the more standardized implementation and higher participant compliance with interventions in institutional settings. For exercise mode, balance training yielded a slightly better effect than multimodal training, which was consistent with its core mechanism of directly improving postural control ability [60]. The absence of significant differences in the above dimensions might be due to the small sample size of each subgroup and the heterogeneity of intervention protocols across studies. The similar effect sizes across other subgroups, including interveners’ qualifications, exposure dosage, and digital platform type, confirmed the robustness of such interventions, providing a reference for their flexible implementation in clinical and community settings.

For concerns about falling, our findings indicate that digital-based exercise interventions did not significantly improve concerns about falling, which is consistent with the findings of Lee et al [28], who used the FES-I. This lack of efficacy may be attributed to 2 primary reasons. First, concerns about falling, as an emotional construct, is fundamentally distinct from the cognitive construct. While a cognitive construct is closely tied to an individual’s assessment of their balance capacity and can thus be improved through objective functional gains, the affective construct is characterized by concerns about falling, manifesting as a feeling of dread and apprehension toward situations perceived as threatening to balance [17]. This nonrational emotional response is resistant to standardized interventions that primarily target physical function alone. Second, there is a mismatch in the active components of the existing interventions. The digital interventions included in this analysis were predominantly based on physical training and lacked elements specifically designed to address worrying emotions, making them mechanistically inadequate for directly targeting the core of emotional constructs.

### Effect of Digital-Based Exercise Interventions on Physical Performance

The pooled results demonstrated that digital-based exercise interventions significantly improved physical performance, including balance function, functional mobility, and physical function, in older adults. These findings are consistent with previous research. Most digital-based interventions implement structured exercise programs, particularly targeted balance training. These exercises have been proven to enhance lower limb strength, postural stability, and sensorimotor integration, which is the key physiological basis for evaluating balance function and activity ability [55,56]. Systematic exercise also improves the integrated physical capacity needed for daily activities, as reflected in physical function. In addition, digital platforms significantly amplify the effects of these exercises. Exergaming and immersive VR mitigate the monotony of traditional training, substantially increasing motivation, engagement, and adherence [61]. Critically, digital delivery overcomes spatial and temporal barriers, enhancing accessibility and convenience [62,63]. This allows for more frequent and consistent participation, enabling exercise benefits to accumulate fully. Furthermore, the improvement outcomes for both the moderate exposure dosage (20‐50 times) and the lower dosage (<20 times) were significantly superior to those of the high dosage (>50 times), suggesting that beyond a certain threshold, further increases in exercise volume may not yield additional functional gains and could even diminish effects due to factors such as fatigue or reduced adherence [64].

### Strengths and Limitations of the Study

This study has several strengths. First, this study provides the first meta-analytic evidence quantifying the effects of digital-based exercise interventions on both the cognitive and emotional constructs of fall-related psychological impairments among older adults, clearly demonstrating their differential effectiveness. Second, the study was rigorous, using comprehensive terminology and an extensive systematic search strategy. Only reliable evidence from RCTs was included, and registered protocols were adhered to, providing more precise effect estimates.

Moreover, our study has several limitations. First, this study included older adults with different baseline characteristics, and the heterogeneity of the population indicated that many subjects differed in terms of their pathophysiological behaviors and conditions, as well as their comorbidities, all of which may have affected the accuracy of the reported results. Second, most of the original studies included in this review were not designed primarily to improve “concerns about falling” or “falls efficacy,” which could lead to an underestimation of the overall effect and an amplification of heterogeneity. The use of self-reported scales to measure fear of falling suggests the possibility of bias and may have led to inaccurate effect size estimates, especially for studies that did not have a blinded assignment to treatment versus control.

### Suggestions for Further Research

While the study demonstrates limited efficacy in improving concerns about falling (an emotional construct), it reveals a more positive effect on falls efficacy (a cognitive construct). These differential outcomes underscore the need for more precisely targeted intervention designs in the future. Therefore, clinicians and researchers should select or design digital interventions on the basis of an individual’s primary presentation.

To develop these more precisely targeted interventions, future research should prioritize the development of digital programs that integrate evidence-based psychological components, such as cognitive behavioral therapy and mindfulness training, to directly target the core mechanisms of emotional constructs. Future studies should employ more refined research designs to further clarify the specific pathways through which different intervention types (exercise, cognitive, and combined) influence distinct constructs. Furthermore, in terms of outcome assessment, in addition to self-report scales, future studies should explore the use of objective physiological biomarkers or behavioral tasks to more sensitively measure changes in emotional constructs.

### Conclusions

The meta-analysis assessed the efficacy of digital-based exercise interventions on fall-related psychological impairments among older adults and revealed that the effects differed across the constructs. These findings suggest that digital-based exercise interventions have potential benefits for improving falls efficacy and physical performance among older adults compared with controls. However, the effect of digital-based exercise interventions on concerns about falling, which is the emotional construct related to falls, remains uncertain among older adults.

## Supplementary material

10.2196/87070 Multimedia Appendix 1 Search strategy; subgroup analyses on balance (Berg Balance Scale), functional mobility (Timed Up and Go Test), and physical function (Short Physical Performance Battery); and funnel plots.

10.2196/87070 Checklist 1 PRISMA checklist.

## Abbreviations

FES–I: Falls Efficacy Scale–International
GRADE: Grading of Recommendations Assessment, Development, and Evaluation
MD: mean difference
MeSH: Medical Subject Headings
MFES: Modified Falls Efficacy Scale
PRISMA: Preferred Reporting Items for Systematic Reviews and Meta-Analyses
RCT: randomized controlled trial
RoB2: Risk of Bias 2
SMD: standardized mean difference
VR: virtual reality
