# Supplementary material for: Effects of Digital-Based Exercise Interventions on Concerns About Falling, Falls Efficacy, and Physical Performance Among Older Adults: Systematic Review and Meta-Analysis
Source: JMIR Aging. 2026 Apr 30;9:e87070. doi: 10.2196/87070 (PMC13132488; doi:10.2196/87070)
Supplement: Multimedia Appendix 1 [file aging-v9-e87070-s001.docx]

| SinoMed |  |
| --- | --- |
| #1 | "老年人"[常用字段:智能] OR "老人"[常用字段:智能] OR "老年患者"[常用字段:智能] OR "老年"[常用字段:智能] OR "老年病人"[常用字段:智能] |
| #2 | "数字健康干预"[常用字段:智能] OR "数字健康"[常用字段:智能] OR "数字干预"[常用字段:智能] OR "数字疗法"[常用字段:智能] OR "数字健康教育"[常用字段:智能] OR "数字健康技术"[常用字段:智能] |
| #3 | "虚拟现实"[常用字段:智能] OR "虚拟现实技术"[常用字段:智能] OR "虚拟仿真"[常用字段:智能] OR "增强现实"[常用字段:智能] OR "VR"[常用字段:智能] OR "虚拟现实场景"[常用字段:智能] OR "AR"[常用字段:智能] OR "虚拟现实暴露疗法"[常用字段:智能] OR "虚拟环境"[常用字段:智能] |
| #4 | "运动游戏"[常用字段:智能] OR "任天堂"[常用字段:智能] OR "运动电玩"[常用字段:智能] OR "体感游戏"[常用字段:智能] |
| #5 | "远程医疗"[常用字段:智能] OR "可穿戴设备"[常用字段:智能] OR "移动应用程序"[常用字段:智能] OR "小程序"[常用字段:智能] OR "APP"[常用字段:智能] OR "移动应用"[常用字段:智能] OR "网络"[常用字段:智能] OR "互联网"[常用字段:智能] |
| #6 | "跌倒恐惧"[常用字段:智能] OR "害怕跌倒"[常用字段:智能] OR "跌倒恐惧感"[常用字段:智能] OR "担心跌倒"[常用字段:智能] OR "跌倒效能"[常用字段:智能] OR "跌倒恐惧程度"[常用字段:智能] |
| #7 | #2 OR #3 OR #4 OR #5 |
| #8 | #1 AND #6 AND #7 |

***Supplementary appendix 1.Search strategy***

***Literature search from build to 2025.05***

| VIPC |  |
| --- | --- |
| #1 | [((((((题名或关键词=老年人 OR 题名或关键词=老年) OR 题名或关键词=老年患者) OR 题名或关键词=老人) OR 题名或关键词=老年病人) AND (((((题名或关键词=跌倒恐惧 OR 题名或关键词=害怕跌倒) OR 题名或关键词=担心跌倒) OR 题名或关键词=跌倒效能) OR 题名或关键词=跌倒恐惧程度) OR 题名或关键词=跌倒恐惧感)) AND ((((((((((((((((((((((((((题名或关键词=数字健康干预 OR 题名或关键词=数字健康技术) OR 题名或关键词=数字技术) OR 题名或关键词=数字疗法) OR 题名或关键词=数字健康教育) OR 题名或关键词=数字干预) OR 题名或关键词=虚拟现实) OR 题名或关键词=虚拟现实技术) OR 题名或关键词=虚拟仿真) OR 题名或关键词=VR技术) OR 题名或关键词=AR) OR 题名或关键词=虚拟现实场景) OR 题名或关键词=增强现实) OR 题名或关键词=虚拟环境) OR 题名或关键词=虚拟现实暴露疗法) OR 题名或关键词=运动游戏) OR 题名或关键词=任天堂) OR 题名或关键词=运动电玩) OR 题名或关键词=体感游戏) OR 题名或关键词=远程医疗) OR 题名或关键词=可穿戴设备) OR 题名或关键词=移动应用程序) OR 题名或关键词=小程序) OR 题名或关键词=app) OR 题名或关键词=移动应用) OR 题名或关键词=网络) OR 题名或关键词=互联网))](https://qikan.cqvip.com/Qikan/search/index?LngMySearHistoryIdGuid=f9332085-fddb-48f2-b157-13671ba849a5&from=Qikan_Article_History) |

| Wanfang databases |  |
| --- | --- |
| #1 | （主题:(老年人) OR 题名或关键词:(老年人 OR 老年 OR 老年患者 OR 老人 OR 老年病人)） AND （主题:(跌倒恐惧) OR 题名或关键词:(跌倒恐惧 OR 害怕跌倒 OR 担心跌倒 OR 跌倒效能 OR 跌倒恐惧程度 OR 跌倒恐惧感)） AND (主题:(数字健康干预 OR 穿戴设备 OR 虚拟现实 OR 远程医疗 OR 互联网 ) OR 题名或关键词:(数字健康干预 OR 数字健康技术 OR 数字技术 OR 数字疗法 OR 数字健康教育 OR 数字干预 OR 虚拟现实 OR 虚拟现实技术 OR 虚拟仿真 OR VR技术 OR AR OR 虚拟现实场景)) |

**CINAHL**

| S12 | S1 AND S9 AND S10 AND S11 |
| --- | --- |
| S11 | (MH "Randomized Controlled Trials") OR SU ( controlled trial, randomized OR randomised controlled stud* OR randomised controlled trial* OR randomized controlled stud* OR trial*, randomized controlled OR randomized controlled trial* OR rct OR Quantitative Studies OR placebo* OR randomi* control* trial* OR Random Assignment OR Clinical Trial* OR allocat* random* OR Quantitative Stud* OR placebo* OR random* allocat* OR singl* n1 blind* OR singl* n1 mask* OR doubl* n1 blind* OR doubl* n1 mask* OR tripl* n1 blind* OR tripl* n1 mask* OR trebl* n1 blind* OR trebl* n1 mask* OR clinic* n1 trial* ) |
| S10 | (MH "Fear") OR SU ( fear of falling OR FOF OR fear of fall* OR fear of walking OR fear of ambulat* OR fall-related anxiety OR fall-related anxiety OR fall-related concer* OR fall-related psychological concern* OR fall* efficacy OR balance confidence OR activities-specific balance confidence OR activity avoidance OR activities avoidance OR fear avoidance OR avoid activities OR activity restriction OR restriction* OR activities restriction ) |
| S9 | S2 OR S3 OR S4 OR S5 OR S6 OR S7 OR S8 |
| S8 | (MH "Internet-Based Intervention") OR SU ( website OR web-based* OR website delivered OR internet OR online* OR tablet OR ipad OR web OR world wide web OR Internet Based Intervention* OR Internet-Based Intervention* OR Intervention*, Internet-Based OR Intervention, Web-based OR Web-based Intervention* OR Internet Intervention* OR Intervention*, Internet OR online intervention OR digital intervention* OR technology-based OR technology based ) |
| S7 | (MH "Mobile Applications") OR SU ( Application, Mobile* OR Mobile Application OR Mobile App OR Mobile Apps OR App, Mobile OR Portable Software App OR App, Portable Software OR Software App, Portable OR Portable Software Application* OR Application, Portable Software OR Software Application, Portable OR Smartphone* OR App, Smartphone OR Portable Electronic App OR App, Portable Electronic OR Electronic App, Portable OR Portable Electronic Application* OR Application, Portable Electronic OR Electronic Application, Portable OR smartwatch* OR smart watch* OR tele monitor* OR telemonitor* OR mobile technolog* OR text message* OR health app OR health apps ) |
| S6 | ( (MH "Telemedicine") OR (MH "Telehealth") ) OR SU ( Tele-Referra* OR Tele Referral OR Virtual Medicine OR Medicine, Virtual OR Telehealth OR eHealth OR Telecare OR Tele-Care OR Tele Care OR Tele-Intensive Care OR Tele Intensive Care OR Tele-ICU OR Tele ICU OR Mobile Health OR Health, Mobile OR mHealth ) |
| S5 | (MH "Exergames") OR SU ( Active-Video Game OR Gaming, Active-Video OR Virtual Reality Exercise* OR Exercise*, Virtual Reality OR exergam* OR videogam* OR wii-fit OR wii fit ) |
| S4 | ( (MH "Virtual Reality") OR (MH "Virtual Reality Exposure Therapy") ) OR SU ( Reality, Virtual OR Virtual Reality, Educational OR Educational Virtual Realit* OR Virtual Realit*, Educational OR Virtual Realit*, Instructional OR Instructional Virtual Realit* OR Realities, Instructional Virtual OR Reality, Instructional Virtual OR digital treatment* OR Virtual Reality Immersion Therapy OR Virtual Reality Therap* OR Reality Therapies, Virtual OR Reality Therapy, Virtual OR Therapies, Virtual Reality OR Therapy, Virtual Reality OR vr exposure therapy OR vr immersion therapy OR vret (virtual reality exposure therapy) OR reality, virtual ) |
| S3 | (MH "Wearable Sensors") OR SU ( Wearable Electronic Devices OR Device, Wearable Electronic OR Electronic Device, Wearable OR Wearable Electronic Device* OR Technology, Wearable OR Wearable Technologie* OR Wearable Device* OR Device, Wearable OR Wearable Computer* OR Computer, Wearable OR Electronic Skin OR Skin, Electronic ) |
| S2 | ( (MH "Digital Health") OR (MH "Digital Technology") ) OR SU ( digital health OR health, digital OR digital health technolog* OR health technologies, digital OR health technology, digital ) |
| S1 | (MH "Aged") OR SU ( Elderly OR senior* OR elderly OR old OR aged* OR older adult* OR elder people OR senior citizens OR old folks OR old people OR aging OR old-aged OR elder* OR senior* OR geriatric* OR oldest old OR oldest-old OR very-old OR septuagenarian* OR octogenarian* OR nonagenarian* OR aged patient OR aged people OR aged person OR aged subject OR elderly patient OR elderly people OR elderly person OR elderly subject OR senior citizen OR senium ) |

**PUBMED**

| #1 | "Aged"[Mesh] |
| --- | --- |
| #2 | (Elderly ) OR ( senior* ) OR ( elderly ) OR ( old ) OR ( aged* ) OR ( older adult* ) OR ( elder people ) OR ( senior citizens ) OR ( old folks ) OR ( old people ) OR ( aging ) OR ( old-aged ) OR ( elder* ) OR ( senior* ) OR ( geriatric* ) OR ( oldest old ) OR ( oldest-old ) OR ( very-old ) OR ( septuagenarian* ) OR ( octogenarian* ) OR ( nonagenarian* ) OR ( aged patient ) OR ( aged people ) OR ( aged person ) OR ( aged subject ) OR ( elderly patient ) OR ( elderly people ) OR ( elderly person ) OR ( elderly subject ) OR ( senior citizen ) OR ( senium') |
| #3 | #1 OR #2 |
| #4 | "Digital Health"[Mesh] |
| #5 | (digital health) OR (health, digital) OR (digital health technolog*) OR (health technologies, digital) OR (health technology, digital) OR (digital intervention) |
| #6 | "Wearable Electronic Devices"[Mesh] |
| #7 | (Device, Wearable Electronic) OR (Electronic Device, Wearable) OR (Wearable Electronic Device*) OR (Technology, Wearable) OR (Wearable Technologie*) OR (Wearable Device*) OR (Device, Wearable) OR (Wearable Computer*) OR (Computer, Wearable) OR (Electronic Skin) OR (Skin, Electronic) |
| #8 | "Virtual Reality"[Mesh] OR "Virtual Reality Exposure Therapy"[Mesh] OR "Exergaming"[Mesh] |
| #9 | (Reality, Virtual)OR(Virtual Reality, Educational)OR (Educational Virtual Realit*) OR (Virtual Realit*, Educational) OR (Virtual Realit*, Instructional) OR (Instructional Virtual Realit*) OR (Realities, Instructional Virtual) OR (Reality, Instructional Virtual) OR (digital treatment*) OR (Virtual Reality Immersion Therapy) OR (Virtual Reality Therap*) OR (Reality Therapies, Virtual) OR (Reality Therapy, Virtual) OR (Therapies, Virtual Reality) OR (Therapy, Virtual Reality) OR (vr exposure therapy) OR (vr immersion therapy) OR (vret (virtual reality exposure therapy)) OR(reality, virtual) OR (Active-Video Game) OR (Gaming, Active-Video) OR (Virtual Reality Exercise*) OR (Exercise*, Virtual Reality) OR (exergam*) OR (videogam*) OR (wii-fit) OR (wii fit) |
| #10 | "Telemedicine"[Mesh] |
| #11 | (Tele-Referra*) OR (Tele Referral) OR (Virtual Medicine) OR (Medicine, Virtual) OR (Telehealth) OR (eHealth) OR (Telecare) OR (Tele-Care) OR (Tele Care) OR (Tele-Intensive Care) OR (Tele Intensive Care)OR (Tele-ICU) OR (Tele ICU) OR (Mobile Health) OR (Health, Mobile) OR (mHealth) |
| #12 | "Mobile Applications"[Mesh] |
| #13 | (Application, Mobile*) OR(Mobile Application) OR (Mobile App) OR (Mobile Apps) OR (App, Mobile) OR (Portable Software App) OR (App, Portable Software) OR (Software App, Portable) OR (Portable Software Application*) OR (Application, Portable Software) OR (Software Application, Portable) OR (Smartphone*) OR (App, Smartphone) OR (Portable Electronic App) OR (App, Portable Electronic) OR (Electronic App, Portable) OR (Portable Electronic Application*) OR (Application, Portable Electronic) OR(Electronic Application, Portable) OR (smartwatch*) OR (smart watch*) OR (tele monitor*) OR (telemonitor*) OR (mobile technolog*) OR (text message*) OR (health app) OR (health apps) |
| #14 | "Internet-Based Intervention"[Mesh] |
| #15 | (website) OR (web-based*) OR (website delivered)OR (internet) OR(online*) OR (tablet) OR (ipad) OR (web) OR (world wide web) OR (Internet Based Intervention*) OR (Internet-Based Intervention*) OR (Intervention*, Internet-Based) OR (Intervention, Web-based) OR (Web-based Intervention*) OR (Internet Intervention*) OR (Intervention*, Internet) OR (online intervention) OR (digital intervention*) OR (technology-based) OR (technology based) |
| #16 | (#4 OR #5 OR #6 OR #7 OR #8 OR #9 OR #10 OR #11 OR #12 OR #13 OR #14 OR #15) |
| #17 | "Fear"[Mesh] |
| #18 | (fear of falling)OR (FOF) OR(fear of fall*) OR (fear of walking) OR (fear of ambulat*) OR (fall-related anxiety) OR (fall-related anxiety) OR (fall-related concer*) OR(fall-related psychological concern*) OR (fall* efficacy) OR (balance confidence) OR (activities-specific balance confidence) OR (activity avoidance) OR (activities avoidance) OR (fear avoidance) OR (avoid activities) OR (activity restriction) OR (restriction*) OR (activities restriction) |
| #19 | "Randomized Controlled Trial" [Publication Type] |
| #20 | (controlled trial, randomized) OR (randomised controlled stud*) OR (randomised controlled trial*) OR (randomized controlled stud*) OR (trial*, randomized controlled) OR (randomized controlled trial*) OR (rct) OR (Quantitative Studies) OR ( placebo*) OR (randomi* control* trial*) OR (Random Assignment) OR (Clinical Trial*) OR (allocat* random*) OR (Quantitative Stud*) OR (placebo*) OR (random* allocat*) OR (singl* n1 blind*) OR (singl* n1 mask*) OR (doubl* n1 blind*) OR (doubl* n1 mask*) OR (tripl* n1 blind*) OR (tripl* n1 mask*) OR (trebl* n1 blind*) OR (trebl* n1 mask*) OR (clinic* n1 trial*) |
| #21 | #19OR #20 |
| #22 | #17 OR #18 |
| #23 | #3 AND #16 AND #21 AND #22 |

**embase**

| #26 | #3 AND #18 AND #22 AND #25 |
| --- | --- |
| #25 | #23 OR #24 |
| #24 | 'controlled trial, randomized'/exp OR 'controlled trial, randomized' OR 'randomised controlled stud*' OR 'randomised controlled trial*' OR 'randomized controlled stud*' OR 'trial*, randomized controlled' OR 'randomized controlled trial*' OR 'rct' OR 'quantitative studies'/exp OR 'quantitative studies' OR 'randomi* control* trial*' OR 'random assignment' OR 'clinical trial*' OR 'allocat* random*' OR 'quantitative stud*' OR 'placebo*' OR 'random* allocat*' OR 'singl* n1 blind*' OR 'singl* n1 mask*' OR 'doubl* n1 blind*' OR 'doubl* n1 mask*' OR 'tripl* n1 blind*' OR 'tripl* n1 mask*' OR 'trebl* n1 blind*' OR 'trebl* n1 mask*' OR 'clinic* n1 trial*' |
| #23 | 'randomized controlled trial'/exp |
| #22 | #19 OR #20 OR #21 |
| #21 | 'fear of falling'/exp OR 'fear of falling' OR 'fof' OR 'fear of fall*' OR 'fear of walking'/exp OR 'fear of walking' OR 'fear of ambulat*' OR 'fall-related anxiety' OR 'fall-related concer*' OR 'fall-related psychological concern*' OR 'fall* efficacy' OR 'balance confidence'/exp OR 'balance confidence' OR 'activities-specific balance confidence'/exp OR 'activities-specific balance confidence' OR 'activity avoidance' OR 'activities avoidance' OR 'fear avoidance'/exp OR 'fear avoidance' OR 'avoid activities' OR 'activity restriction'/exp OR 'activity restriction' OR 'restriction*' OR 'activities restriction' |
| #20 | 'fear'/exp |
| #19 | 'fear of falling'/exp |
| #18 | #4 OR #5 OR #6 OR #7 OR #8 OR #9 OR #10 OR #11 OR #12 OR #13 OR #14 OR #15 OR #16 OR #17 |
| #17 | 'website'/exp OR 'website' OR 'web-based*' OR 'website delivered' OR 'internet'/exp OR 'internet' OR 'online*' OR 'tablet'/exp OR 'tablet' OR 'ipad'/exp OR 'ipad' OR 'web'/exp OR 'web' OR 'world wide web'/exp OR 'world wide web' OR 'internet based intervention*' OR 'internet-based intervention*' OR 'intervention*, internet-based' OR 'intervention, web-based' OR 'web-based intervention*' OR 'internet intervention*' OR 'intervention*, internet' OR 'online intervention'/exp OR 'online intervention' OR 'digital intervention*' OR 'technology-based' OR 'technology based' |
| #16 | 'web-based intervention'/exp |
| #15 | 'application, mobile*' OR 'mobile application' OR 'mobile app'/exp OR 'mobile app' OR 'mobile apps'/exp OR 'mobile apps' OR 'app, mobile' OR 'portable software app'/exp OR 'portable software app' OR 'app, portable software' OR 'software app, portable' OR 'portable software application*' OR 'application, portable software' OR 'software application, portable' OR 'smartphone*' OR 'app, smartphone' OR 'portable electronic app' OR 'app, portable electronic' OR 'electronic app, portable' OR 'portable electronic application*' OR 'application, portable electronic' OR 'electronic application, portable' OR 'smartwatch*' OR 'smart watch*' OR 'tele monitor*' OR 'telemonitor*' OR 'mobile technolog*' OR 'text message*' OR 'health app' OR 'health apps' |
| #14 | 'mobile application'/exp |
| #13 | 'tele-referra*' OR 'tele referral' OR 'virtual medicine'/exp OR 'virtual medicine' OR 'medicine, virtual' OR 'telehealth'/exp OR 'telehealth' OR 'ehealth'/exp OR 'ehealth' OR 'telecare'/exp OR 'telecare' OR 'tele-care'/exp OR 'tele-care' OR 'tele care'/exp OR 'tele care' OR 'tele-intensive care' OR 'tele intensive care' OR 'tele-icu' OR 'tele icu' OR 'mobile health'/exp OR 'mobile health' OR 'health, mobile' OR 'mhealth'/exp OR 'mhealth' |
| #12 | 'telemedicine'/exp |
| #11 | 'active-video game' OR 'gaming, active-video' OR 'virtual reality exercise*' OR 'exercise*, virtual reality' OR 'exergam*' OR 'videogam*' OR 'wii-fit' OR 'wii fit'/exp OR 'wii fit' |
| #10 | 'exergaming'/exp |
| #9 | 'virtual reality, educational' OR 'educational virtual realit*' OR 'virtual realit*, educational' OR 'virtual realit*, instructional' OR 'instructional virtual realit*' OR 'realities, instructional virtual' OR 'reality, instructional virtual' OR 'digital treatment*' OR 'virtual reality immersion therapy' OR 'virtual reality therap*' OR 'reality therapies, virtual' OR 'reality therapy, virtual' OR 'therapies, virtual reality' OR 'therapy, virtual reality or vr exposure therapy' OR 'vr immersion therapy'/exp OR 'vr immersion therapy' OR 'vret (virtual reality exposure therapy)'/exp OR 'vret (virtual reality exposure therapy)' OR 'reality, virtual' |
| #8 | 'virtual reality'/exp OR 'virtual reality exposure therapy'/exp |
| #7 | 'device, wearable electronic' OR 'electronic device, wearable' OR 'wearable electronic device*' OR 'technology, wearable' OR 'wearable technologie*' OR 'wearable device*' OR 'device, wearable' OR 'wearable computer*' OR 'computer, wearable' OR 'electronic skin'/exp OR 'electronic skin' OR 'skin, electronic' |
| #6 | 'wearable computer'/exp |
| #5 | 'digital health'/exp OR 'digital health' OR 'health, digital' OR 'digital health technolog*' OR 'health technologies, digital' OR 'health technology, digital' |
| #4 | 'digital health'/exp OR 'digital health technology'/exp |
| #3 | #1 OR #2 |
| #2 | 'elderly'/exp OR 'elderly' OR 'old' OR 'aged*' OR 'older adult*' OR 'elder people' OR 'senior citizens' OR 'old folks' OR 'old people' OR 'aging'/exp OR 'aging' OR 'old-aged' OR 'elder*' OR 'senior*' OR 'geriatric*' OR 'oldest old' OR 'oldest-old' OR 'very-old'/exp OR 'very-old' OR 'septuagenarian*' OR 'octogenarian*' OR 'nonagenarian*' OR 'aged patient'/exp OR 'aged patient' OR 'aged people'/exp OR 'aged people' OR 'aged person'/exp OR 'aged person' OR 'aged subject'/exp OR 'aged subject' OR 'elderly patient'/exp OR 'elderly patient' OR 'elderly people'/exp OR 'elderly people' OR 'elderly person'/exp OR 'elderly person' OR 'elderly subject'/exp OR 'elderly subject' OR 'senior citizen'/exp OR 'senior citizen' OR 'senium'/exp OR 'senium' |
| #1 | 'aged'/exp |

**Cochrane**

| #1 | MeSH descriptor: [Aged] explode all trees |
| --- | --- |
| #2 | (Elderly OR senior* OR elderly OR old OR aged* OR older adult* OR elder people OR senior citizens OR old folks OR old people OR aging OR old-aged OR elder* OR senior* OR geriatric* OR oldest old OR oldest-old OR very-old OR septuagenarian* OR octogenarian* OR nonagenarian*):ti,ab,kw (Word variations have been searched) |
| #3 | #1 OR #2 |
| #4 | MeSH descriptor: [Digital Health] explode all trees |
| #5 | (digital health OR health, digital OR digital health technolog* OR health technologies, digital OR health technology, digital):ti,ab,kw (Word variations have been searched) |
| #6 | MeSH descriptor: [Wearable Electronic Devices] explode all trees |
| #7 | (Device, Wearable Electronic OR Electronic Device, Wearable OR Wearable Electronic Device* OR Technology, Wearable OR Wearable Technologie* OR Wearable Device* OR Device, Wearable OR Wearable Computer* OR Computer, Wearable OR Electronic Skin OR Skin, Electronic):ti,ab,kw (Word variations have been searched) |
| #8 | MeSH descriptor: [Virtual Reality] explode all trees |
| #9 | MeSH descriptor: [Virtual Reality Exposure Therapy] explode all trees |
| #10 | (Reality, Virtual OR Virtual Reality, Educational OR Educational Virtual Realit* OR Virtual Realit*, Educational OR Virtual Realit*, Instructional OR Instructional Virtual Realit* OR Realities, Instructional Virtual OR Reality, Instructional Virtual OR digital treatment* OR Virtual Reality Immersion Therapy OR Virtual Reality Therap* OR Reality Therapies, Virtual OR Reality Therapy, Virtual OR Therapies, Virtual Reality OR Therapy, Virtual Reality OR vr exposure therapy OR vr immersion therapy OR vret (virtual reality exposure therapy) OR reality, virtual):ti,ab,kw (Word variations have been searched) |
| #11 | MeSH descriptor: [Exergaming] explode all trees |
| #12 | (Active-Video Game OR Gaming, Active-Video OR Virtual Reality Exercise* OR Exercise*, Virtual Reality OR exergam* OR videogam* OR wii-fit OR wii fit):ti,ab,kw (Word variations have been searched) |
| #13 | MeSH descriptor: [Telemedicine] explode all trees |
| #14 | (Tele-Referra* OR Tele Referral OR Virtual Medicine OR Medicine, Virtual OR Telehealth OR eHealth OR Telecare OR Tele-Care OR Tele Care OR Tele-Intensive Care OR Tele Intensive Care OR Tele-ICU OR Tele ICU OR Mobile Health OR Health, Mobile OR mHealth):ti,ab,kw (Word variations have been searched) |
| #15 | MeSH descriptor: [Mobile Applications] explode all trees |
| #16 | (Application, Mobile* OR Mobile Application OR Mobile App OR Mobile Apps OR App, Mobile OR Portable Software App OR App, Portable Software OR Software App, Portable OR Portable Software Application* OR Application, Portable Software OR Software Application, Portable OR Smartphone* OR App, Smartphone OR Portable Electronic App OR App, Portable Electronic OR Electronic App, Portable OR Portable Electronic Application* OR Application, Portable Electronic OR Electronic Application, Portable OR smartwatch* OR smart watch* OR tele monitor* OR telemonitor* OR mobile technolog* OR text message* OR health app OR health apps):ti,ab,kw (Word variations have been searched) |
| #17 | MeSH descriptor: [Internet-Based Intervention] explode all trees |
| #18 | (website OR web-based* OR website delivered OR internet OR online* OR tablet OR ipad OR web OR world wide web OR Internet Based Intervention* OR Internet-Based Intervention* OR Intervention*, Internet-Based OR Intervention, Web-based OR Web-based Intervention* OR Internet Intervention* OR Intervention*, Internet OR online intervention OR digital intervention* OR technology-based OR technology based):ti,ab,kw (Word variations have been searched) |
| #19 | #4 OR #5 OR #6 OR #7 OR #8 OR #9 OR #10 OR #11 OR #12 OR #13 OR #14 OR #15 OR #16 OR #17 OR #18 |
| #20 | MeSH descriptor: [Fear] explode all trees |
| #21 | (fear of falling OR FOF OR fear of fall* OR fear of walking OR fear of ambulat* OR fall-related anxiety OR fall-related anxiety OR fall-related concer* OR fall-related psychological concern* OR fall* efficacy OR balance confidence OR activities-specific balance confidence OR activity avoidance OR activities avoidance OR fear avoidance OR avoid activities OR activity restriction OR restriction* OR activities restriction):ti,ab,kw (Word variations have been searched) |
| #22 | (controlled trial, randomized OR randomised controlled stud* OR randomised controlled trial* OR randomized controlled stud* OR trial*, randomized controlled OR randomized controlled trial* OR rct OR Quantitative Studies OR placebo* OR randomi* control* trial* OR Random Assignment OR Clinical Trial* OR allocat* random* OR Quantitative Stud* OR placebo* OR random* allocat* OR singl* n1 blind* OR singl* n1 mask* OR doubl* n1 blind* OR doubl* n1 mask* OR tripl* n1 blind* OR tripl* n1 mask* OR trebl* n1 blind* OR trebl* n1 mask* OR clinic* n1 trial* OR randomised controlled study OR random*):ti,ab,kw (Word variations have been searched) |
| #23 | #20 OR #21 |
| #24 | #3 AND #19 AND #23 AND #22 |

**WOS**

| #1 | TS=(Aged OR Elderly OR senior* OR elderly OR old OR aged* OR older adult* OR elder people OR senior citizens OR old folks OR old people OR aging OR old-aged OR elder* OR senior* OR geriatric* OR oldest old OR oldest-old OR very-old OR septuagenarian* OR octogenarian* OR nonagenarian* OR aged patient OR aged people OR aged person OR aged subject OR elderly patient OR elderly people OR elderly person OR elderly subject OR senior citizen OR senium) |
| --- | --- |
| #2 | TS=(digital health OR digital health technology OR digital intervention OR digital health OR health, digital OR digital health technolog* OR health technologies, digital OR health technology, digital) |
| #3 | TS=(Wearable Electronic Devices OR Device, Wearable Electronic OR Electronic Device, Wearable OR Wearable Electronic Device* OR Technology, Wearable OR Wearable Technologie* OR Wearable Device* OR Device, Wearable OR Wearable Computer* OR Computer, Wearable OR Electronic Skin OR Skin, Electronic) |
| #4 | TS=(Virtual Reality OR Virtual Reality Exposure Therapy OR Reality, Virtual OR Virtual Reality, Educational OR Educational Virtual Realit* OR Virtual Realit*, Educational OR Virtual Realit*, Instructional OR Instructional Virtual Realit* OR Realities, Instructional Virtual OR Reality, Instructional Virtual OR digital treatment* OR Virtual Reality Immersion Therapy OR Virtual Reality Therap* OR Reality Therapies, Virtual OR Reality Therapy, Virtual OR Therapies, Virtual Reality OR Therapy, Virtual Reality OR vr exposure therapy OR vr immersion therapy OR vret (virtual reality exposure therapy) OR reality, virtual) |
| #5 | TS=(Exergaming OR exergames OR Active-Video Game OR Gaming, Active-Video OR Virtual Reality Exercise* OR Exercise*, Virtual Reality OR exergam* OR videogam* OR wii-fit OR wii fit) |
| #6 | TS=(Telemedicine OR Tele-Referra* OR Tele Referral OR Virtual Medicine OR Medicine, Virtual OR Telehealth OR eHealth OR Telecare OR Tele-Care OR Tele Care OR Tele-Intensive Care OR Tele Intensive Care OR Tele-ICU OR Tele ICU OR Mobile Health OR Health, Mobile OR mHealth) |
| #7 | TS=(Mobile Applications OR Application, Mobile* OR Mobile Application OR Mobile App OR Mobile Apps OR App, Mobile OR Portable Software App OR App, Portable Software OR Software App, Portable OR Portable Software Application* OR Application, Portable Software OR Software Application, Portable OR Smartphone* OR App, Smartphone OR Portable Electronic App OR App, Portable Electronic OR Electronic App, Portable OR Portable Electronic Application* OR Application, Portable Electronic OR Electronic Application, Portable OR smartwatch* OR smart watch* OR tele monitor* OR telemonitor* OR mobile technolog* OR text message* OR health app OR health apps) |
| #8 | TS=(website OR web-based* OR website delivered OR internet OR online* OR tablet OR ipad OR web OR world wide web OR Internet Based Intervention* OR Internet-Based Intervention* OR Intervention*, Internet-Based OR Intervention, Web-based OR Web-based Intervention* OR Internet Intervention* OR Intervention*, Internet OR online intervention OR digital intervention* OR technology-based OR technology based OR Internet-Based Intervention) |
| #9 | TS=(Fear OR fear of falling OR FOF OR fear of fall* OR fear of walking OR fear of ambulat* OR fall-related anxiety OR fall-related anxiety OR fall-related concer* OR fall-related psychological concern* OR fall* efficacy OR balance confidence OR activities-specific balance confidence OR activity avoidance OR activities avoidance OR fear avoidance OR avoid activities OR activity restriction OR restriction* OR activities restriction) |
| #10 | TS=(randomised controlled study OR controlled trial, randomized OR randomised controlled stud* OR randomised controlled trial* OR randomized controlled stud* OR trial*, randomized controlled OR randomized controlled trial* OR rct OR Quantitative Studies OR placebo* OR randomi* control* trial* OR Random Assignment OR Clinical Trial* OR allocat* random* OR Quantitative Stud* OR placebo* OR random* allocat* OR singl* n1 blind* OR singl* n1 mask* OR doubl* n1 blind* OR doubl* n1 mask* OR tripl* n1 blind* OR tripl* n1 mask* OR trebl* n1 blind* OR trebl* n1 mask* OR clinic* n1 trial*) |
| #11 | #2 OR #3 OR #4 OR #5 OR #6 OR #7 OR #8 |
| #12 | #11 AND #9 AND #1 |
| #13 | #10 AND #12 |

| CNKI |  |
| --- | --- |
| #1 | (老年人 OR 老年 OR 老年患者 OR 老人 OR 老年病人) AND(跌倒恐惧 OR 害怕跌倒 OR 担心跌倒 OR 跌倒效能 OR 跌倒恐惧程度 OR 跌倒恐惧感) AND(数字健康干预 OR 数字健康技术 OR 数字技术 OR 数字疗法 OR 数字健康教育 OR 数字干预 OR 虚拟现实 OR 虚拟现实技术 OR 虚拟仿真 OR VR技术 OR AR OR 虚拟现实场景 OR [增强现实](javascript:void(0);) OR 虚拟环境 OR 虚拟现实暴露疗法 OR 运动游戏 OR 任天堂 OR 运动电玩 OR 体感游戏 OR 远程医疗 OR 可穿戴设备 OR 移动应用程序 OR 小程序 OR app OR 移动应用 OR 网络 OR 互联网 ) |

PsycINFO

| #1 | SU(Age OR Elderly OR senior* OR elderly OR old OR aged* OR older adult* OR elder people OR senior citizens OR old folks OR old people OR aging OR old-aged OR elder* OR senior* OR geriatric* OR oldest old OR oldest-old OR very-old OR septuagenarian* OR octogenarian* OR nonagenarian* OR aged patient OR aged people OR aged person OR aged subject OR elderly patient OR elderly people OR elderly person OR elderly subject OR senior citizen OR senium) |
| --- | --- |
| #2 | SU(digital health OR digital health technology OR digital intervention OR digital health OR health, digital OR digital health technolog* OR health technologies, digital OR health technology, digital OR Wearable Electronic Devices OR Device, Wearable Electronic OR Electronic Device, Wearable OR Wearable Electronic Device* OR Technology, Wearable OR Wearable Technologie* OR Wearable Device* OR Device, Wearable OR Wearable Computer* OR Computer, Wearable OR Electronic Skin OR Skin, Electronic OR Reality, Virtual OR Virtual Reality, Educational OR Educational Virtual Realit* OR Virtual Realit*, Educational OR Virtual Realit*, Instructional OR Instructional Virtual Realit* OR Realities, Instructional Virtual OR Reality, Instructional Virtual OR digital treatment* OR Virtual Reality Immersion Therapy OR Virtual Reality Therap* OR Reality Therapies, Virtual OR Reality Therapy, Virtual OR Therapies, Virtual Reality OR Therapy, Virtual Reality OR vr exposure therapy OR vr immersion therapy OR vret (virtual reality exposure therapy) OR reality, virtual OR Virtual Reality OR Virtual Reality Exposure Therapy OR Exergaming OR exergames OR Active-Video Game OR Gaming, Active-Video OR Virtual Reality Exercise* OR Exercise*, Virtual Reality OR exergam* OR videogam* OR wii-fit OR wii fit OR Telemedicine OR Tele-Referra* OR Tele Referral OR Virtual Medicine OR Medicine, Virtual OR Telehealth OR eHealth OR Telecare OR Tele-Care OR Tele Care OR Tele-Intensive Care OR Tele Intensive Care OR Tele-ICU OR Tele ICU OR Mobile Health OR Health, Mobile OR mHealth OR Mobile Applications OR Application, Mobile* OR Mobile Application OR Mobile App OR Mobile Apps OR App, Mobile OR Portable Software App OR App, Portable Software OR Software App, Portable OR Portable Software Application* OR Application, Portable Software OR Software Application, Portable OR Smartphone* OR App, Smartphone OR Portable Electronic App OR App, Portable Electronic OR Electronic App, Portable OR Portable Electronic Application* OR Application, Portable Electronic OR Electronic Application, Portable OR smartwatch* OR smart watch* OR tele monitor* OR telemonitor* OR mobile technolog* OR text message* OR health app OR health apps OR Internet-Based Intervention OR web-based intervention OR website OR web-based* OR website delivered OR internet OR online* OR tablet OR ipad OR web OR world wide web OR Internet Based Intervention* OR Internet-Based Intervention* OR Intervention*, Internet-Based OR Intervention, Web-based OR Web-based Intervention* OR Internet Intervention* OR Intervention*, Internet OR online intervention OR digital intervention* OR technology-based OR technology based OR ) |
| #3 | SU(Fear OR fear of falling OR fear of falling OR FOF OR fear of fall* OR fear of walking OR fear of ambulat* OR fall-related anxiety OR fall-related anxiety OR fall-related concer* OR fall-related psychological concern* OR fall* efficacy OR balance confidence OR activities-specific balance confidence OR activity avoidance OR activities avoidance OR fear avoidance OR avoid activities OR activity restriction OR restriction* OR activities restriction) |
| #4 | SU(andomised controlled study OR controlled trial, randomized OR randomised controlled stud* OR randomised controlled trial* OR randomized controlled stud* OR trial*, randomized controlled OR randomized controlled trial* OR rct OR Quantitative Studies OR placebo* OR randomi* control* trial* OR Random Assignment OR Clinical Trial* OR allocat* random* OR Quantitative Stud* OR placebo* OR random* allocat* OR singl* n1 blind* OR singl* n1 mask* OR doubl* n1 blind* OR doubl* n1 mask* OR tripl* n1 blind* OR tripl* n1 mask* OR trebl* n1 blind* OR trebl* n1 mask* OR clinic* n1 trial* ) |
| #5 | #1 AND #2 AND #3 AND #4 |

***Supplementary appendix 2***

***Table S1. Subgroup analysis on Balance (BBS), Functional mobility (TUG), and Physical function (SPPB).***

| **Outcome** | **Variable** | | **Numbers of comparisons** | **Meta-analysis results** | **Heterogeneity** | **Heterogeneity between groups** |
| --- | --- | --- | --- | --- | --- | --- |
| **Balance (BBS)** | Setting | Community | 2 | 5.10(-0.98,11.18) | I²=88.6%,P=0.003 | P=0.929 |
|  |  | Senior care Facilities | 2 | 3.87(1.95,5.80) | I²=49.4%,P=0.160 |  |
|  |  | Hospital | 2 | 4.09(2.07,6.11) | I²=17.4%,P=0.271 |  |
|  | Qualifications of interveners | Non-professional physiotherapist | 4 | 4.22(2.15,6.30) | I²=74.4%,P=0.008 | P=0.831 |
|  |  | Physiotherapist | 2 | 3.87(1.43,6.32) | I²=46.6%, P=0.171 |  |
|  | Exercise mode | Balance-inclusive multimodal Training | 3 | 3.36(1.76,4.95) | I²=58.6%, P=0.089 | P=0.425 |
|  |  | Balance training | 2 | 5.43(-0.03,10.89) | I²=84.2%, P=0.012 |  |
|  |  | Non-balance training | 1 | 5.47(2.33,8.61) | I²=0.0%, P<0.001 |  |
|  | Duration of the intervention | <12weeks | 4 | 3.57(1.77,5.37) | I²=66.3%, P=0.031 | P=0.242 |
|  |  | ≥12weeks | 2 | 5.03(3.38,6.68) | I²=0.0%, P=0.746 |  |
|  | Exposure dosage | <20 | 2 | 5.43(-0.03,10.89) | I²=84.2%, P=0.012 | ***P=0.036*** |
|  |  | 20-50 | 2 | 2.53(1.43,3.63) | I²=0.0%, P=0.417 |  |
|  |  | >50 | 2 | 5.03(3.38,6.68) | I²=0.0%, P=0.746 |  |
| **Functional mobility (TUG)** | Setting | Community | 5 | -1.03 (-1.2, 0.07) | I²=87.5%, P<0.001 | P=0.082 |
|  |  | Senior care facilities | 3 | -2.89 (-4.11, -1.68) | I²=34.9%, P=0.215 |  |
|  |  | Hospital | 2 | -1.80 (-2.34, -1.26) | I²=0.0%, P=0.422 |  |
|  | Qualifications of interveners | Non-professional physiotherapist | 5 | -1.93 (-3.38, -0.47) | I²=89.4%, P<0.001 | P=0.582 |
|  |  | Physiotherapist | 5 | -1.36 (-2.75, 0.03) | I²=89.5%, P<0.001 |  |
|  | Exercise mode | Balance-inclusive multimodal Training | 6 | -1.29 (-2.22, -0.37) | I²=80.1%, P<0.001 | ***P=0.010*** |
|  |  | Balance training | 2 | -1.89 (-5.32, 1.53) | I²=95.8%, P<0.001 |  |
|  |  | Non-balance Training | 1 | -3.62 (-4.81, -2.43) | I²=0%, P<0.001 |  |
|  | Digital platform | Exergaming | 8 | -1.71 (-2.74, -0.68) | I²=90.0%, P<0.001 | P=0.796 |
|  |  | Application | 2 | -1.47 (-2.97, 0.04) | I²=66.1%, P=0.086 |  |
|  | Duration of the intervention | <12weeks | 4 | -2.30 (-3.33, -1.28) | I²=65.0%, P=0.036 | P=0.146 |
|  |  | ≥12weeks | 6 | -1.20 (-2.28, -0.11) | I²=88.1%, P<0.001 |  |
|  | Exposure dosage | <20 | 3 | -1.69 (-3.46, 0.08) | I²=94.3%, P<0.001 | P=0.886 |
|  |  | 20-50 | 3 | -2.08 (-3.12, -1.04) | I²=0.0%, P=0.433 |  |
|  |  | >50 | 4 | -1.62 (-3.24, 0.00) | I²=91.3%, P<0.001 |  |
| **Physical function (SPPB)** | Setting | Community | 3 | 0.24 (-0.16, 0.64) | I²=64.5%, P=0.060 | ***P=0.035*** |
|  |  | Senior care facilities | 1 | 1.32 (0.21, 2.43) | I²=0.0%, P<0.001 |  |
|  |  | Hospital | 2 | 1.13 (0.42, 1.83) | I²=0.0%, P=0.343 |  |
|  | Qualifications of interveners | Non-professional physiotherapist | 2 | 0.42 (-0.30, 1.14) | I²=78.3%, P=0.032 | P=0.522 |
|  |  | Physiotherapist | 4 | 0.78 (-0.04, 1.60) | I²=74.2%, P=0.009 |  |
|  | Digital platform | Exergaming | 2 | 0.14 (-0.16, 0.44) | I²=0.9%, P=0.315 | P=0.077 |
|  |  | VR | 1 | 1.32 (0.21, 2.43) | I²=0.0%, P<0.001 |  |
|  |  | Application | 3 | 0.67 (-0.14, 1.49) | I²=81.5%, P=0.005 |  |
|  | Duration of the intervention | <12weeks | 2 | 0.81 (0.27, 1.34) | I²=0.0%, P=0.831 | P=0.430 |
|  |  | ≥12weeks | 4 | 0.50 (-0.06, 1.05) | I²=75.4%, P=0.007 |  |
|  | Exposure dosage | <20 | 1 | 0.70 (-0.43, 1.83) | I²=0.0%, P<0.001 | P=0.057 |
|  |  | 20-50 | 3 | 0.21 (-0.22, 0.64) | I²=59.1%, P=0.087 |  |
|  |  | >50 | 2 | 1.02 (0.51, 1.53) | I²=2.0%, P=0.312 |  |

***Supplementary appendix 3.Funnel plot.***


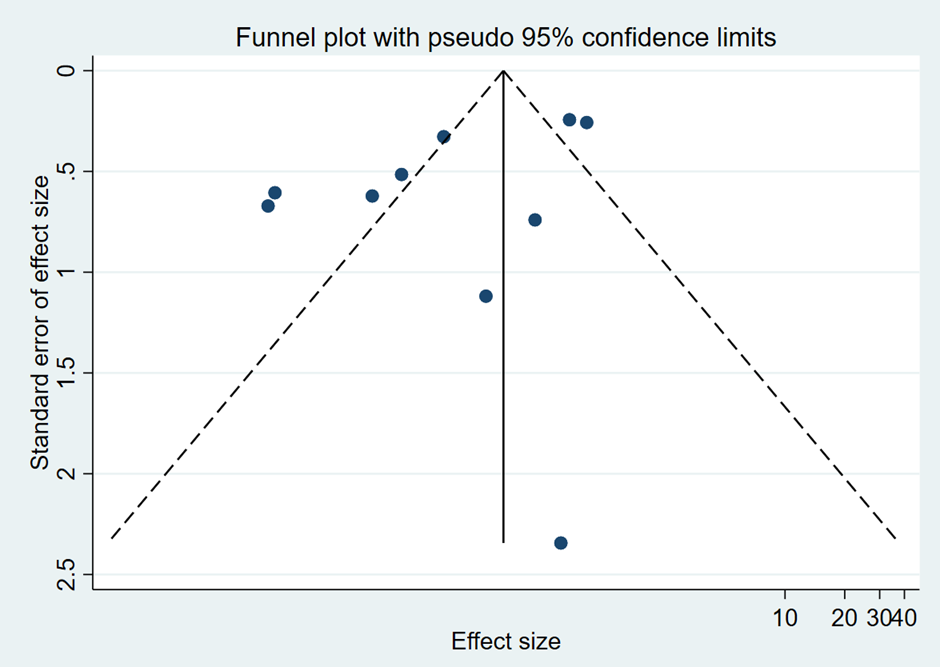


Figure S1. The Funnel plot on functional mobility.
